# Cardiac Traumatism

**Published:** 1916-04

**Authors:** 


**Cardiac Traumatism.** Beaussenat, Acad, de Med. de Paris, reports a case in a soldier aged about 25, struck by a fragment of a hand grenade, which pierced the diaphragm, and pericardium and lodged in the right ventricle, Oct. 1, 1914. Feb. 17, 1915, the fragment was removed and the heart sutured. Intense dyspnoea and imminent syncope were noted for three days, during the next three days there was some fever and there were some pulmonary complications. Recovery was perfect after a month.

